# Measurement properties of the Brazilian online version of the Fibromyalgia Rapid Screening Tool (FiRST)

**DOI:** 10.1186/s42358-022-00271-2

**Published:** 2022-10-31

**Authors:** Ana Paula de Sousa, Guilherme Tavares de Arruda, André Pontes-Silva, Marcelo Cardoso de Souza, Patricia Driusso, Mariana Arias Avila

**Affiliations:** 1 Physical Therapy Department, Universidade Federal de São Carlos, Rod. Washington Luis, km 235, Jardim Guanabara, ZIP Code 13565-905, São Carlos, SP Brazil; 2 Study Group on Chronic Pain (NEDoC), Laboratory of Research on Electrophysical Agents (LAREF), Physical Therapy Department, Universidade Federal de São Carlos, Rod. Washington Luis, km 235, Jardim Guanabara, ZIP Code 13565-905, São Carlos, SP Brazil; 3 Postgraduate Program in Rehabilitation Sciences, Department of Physical Therapy, Universidade Federal do Rio Grande do Norte, Natal, RN Brazil; 4 Laboratory of Research on Women’s Health (LAMU), Physical Therapy Department, Universidade Federal de São Carlos, Rod. Washington Luis, km 235, Jardim Guanabara, ZIP Code 13565-905, São Carlos, SP Brazil

**Keywords:** Chronic pain, Validation study, Fibromyalgia, Surveys and questionnaires

## Abstract

**Objective:**

The Fibromyalgia Rapid Screening Tool (FiRST) was developed to screen people with chronic pain for Fibromyalgia (FM), especially in primary health care settings. This study aimed to translate the FiRST into Brazilian Portuguese and evaluate its measurement properties for an online application.

**Methods:**

After the process of translation and backtranslation, the FiRST was applied online in 483 adults with chronic pain (FM group n = 395; Chronic pain group n = 88), along with the Numerical Rating Scale for pain and fatigue, the Brief Pain Inventory, and the Fibromyalgia Impact Questionnaire-Revised. A Receiver Operating Characteristics (ROC) curve was computed and the area under the curve (AUC) was used to determine the sensibility, specificity, and cut-off score for the FiRST. The Mann-Whitney test was used for quantitative variables and the Chi-square and the Fisher’s exact test, for the categorical variables with level of significance of 5%. Fleiss’ Kappa, Gwet’s AC1 and percentage of agreement were also calculated between test and retest.

**Results:**

For all the questionnaires, the FM group presented higher scores, which mean a worst condition. The FiRST presented a sensitivity of 92.3%, and a specificity of 61.6% with 5 as the cut-off score. AUC, Fleiss’ Kappa, Gwet’s AC1 and percentage of agreement were, respectively, 0.82, 0.38, 0.63 and 71.8%.

**Conclusion:**

The FiRST was translated into Brazilian Portuguese and the online version presented a good content validity and adequate measurement errors that allow FM patients to be screened among people with chronic pain.

## Prior presentation

A part of this manuscript has been presented at the 15th Brazilian Congress on Pain, held virtually September 4th to 7th 2021, with the title “Fibromyalgia Rapid Screening Tool e o rastreio de pacientes com fibromialgia”.

## Significance and innovations

- The Fibromyalgia Rapid Screening Tool (FiRST) was translated into Brazilian Portuguese and presented a good content validity and adequate measurement errors.
- The Brazilian FiRST was collected online and compared with the 2016 American College of Rheumatology fibromyalgia diagnostic criteria and obtained good results.
- The FiRST can also be used as an online tool to screen for fibromyalgia patients.

## Introduction

Fibromyalgia (FM) is a chronic pain condition that affects about 2% of people worldwide [1] and its most common symptoms are the widespread pain, chronic fatigue, and sleep disorders, among others [2]. FM can occur as a primary disease or secondary issue to another existing condition [3]. Due to the lack of clinical exams that can provide evidence of its existence, FM is diagnosed late due to the difficulty in applying the criteria developed by the American College of Rheumatology (ACR) and the complexity of the symptoms. According to the ACR, basically relying on the patients’ self-report; generalized pain, defined as pain in at least 4 of 5 regions; symptoms have been present at a similar level for at least 3 months; widespread pain index (WPI) ≥ 7 and symptom severity scale (SSS) score ≥ 5 (OR WPI of 4–6 and SSS score ≥ 9) [3].

Considering that the difficulty in diagnosing FM impacts on the number of medical appointments, exams, and medications [4], some instruments were developed to add to the screening suggested by the ACR (e.g., the “London Fibromyalgia Epidemiology Study Screening Questionnaire [5]” and the “Regional Pain Scale [6]”). Among those instruments, there is the Fibromyalgia Rapid Screening Tool (FiRST®) by Perrot et al. [7], composed of six items with answer options (yes or no) regarding the main features related to FM, such as pain, fatigue, and sleep. However, this instrument was not translated into Brazilian Portuguese, and nor has it had its measurement properties evaluated.

Usually, the first contact a patient makes with the healthcare system occurs through primary health care, responsible for the assessment, treatment, and in needed, referral of that patient [8], having tools, especially online, that help improve FM diagnosis while shortening the time it takes until the final diagnosis is helpful and may lead to better FM treatment. As such, the aim of the present study was to translate and assess the measurement properties of the online version of the Brazilian FiRST®.

## Methods

This is an instrument validation online study that assessed people with chronic pain and FM. The study was entirely conducted online due to the COVID-19 pandemics and period of social isolation from November 2020 to August 2021. The protocol has been approved by the institutional ethics committee of Universidade Federal de São Carlos (CAAE: 10896719.0.0000.5504, protocol number 4.193.940). The present study was performed according to the Guidelines for the Process of Cross-cultural Adaptation of Self-Report Measures [9] and the COnsensus-based Standards for the Selection of health Measurement INstruments (COSMIN) [10]. Because FiRST® is a screening tool, we evaluated the content validity (degree to which the content of as instrument is an adequate reflection of the construct being measured), test-retest reliability (degree to which a measurement is free from measurement errors over time) and measurement errors (systematic and random error in a patient’s score that is not attributed to actual changes in the construct being measured) of the FiRST® [11].

The study was disclosed in social medias (such as Instagram® and Facebook®) and through messaging application (WhatsApp®). All people who manifested interest in taking part in the study were contacted and checked for eligibility criteria. All those who were included in the study received an online form (via GoogleForms®) and agreed to take part in the study by clicking on the “I agree to take part in the present study” after reading the informed online consent form. All participants received an online booklet with information regarding FM / chronic pain after the end of their participation.

Participants over 18 years old that could read and write in Brazilian Portuguese were included if they presented report of chronic pain (≥ 3 months) and after that, they were divided into two groups: FM group and chronic pain group. For the FM group, people should have the FM diagnosis (participants were considered as with FM if they fulfilled the ACR 2016 FM diagnostic criteria [3], including the WPI ≥ 7 and the SSS ≥ 5 or WPI = 4–6 and SSS ≥ 9). For the chronic pain group, participants should have a history of chronic pain (> 3 months), but no FM. Participants were excluded from the analysis if they had history of tumors, traumas or acute infections and self-report of severe psychiatric illnesses, including severe depression, bipolarity and schizophrenia.

Initially, we obtained authorization to use FiRST® by the MAPI Research Trust (ID 109,594). The translation process occurred according to the recommendations of Beaton et al. [9] in 5 different steps: (1) translation; (2) consensus of the translated version; (3) backtranslation; (4) consensus on the backtranslation; (5) development of the version to be tested. The translation was performed by three Brazilian Portuguese speakers with fluency in English, in which two of them were physical therapists and other was a healthcare professional. After the consensus, the translated version was backtranslated by two native English speakers with fluency on Brazilian Portuguese, one of them not related to a healthcare profession. After the second consensus, the content validity of the FiRST® was tested by 10 healthcare professionals (3 physicians, 6 physical therapists and 1 nurse), all of which have experience with Rheumatology patients, especially FM. After this assessment, a sample of 20 women with FM tested the final FiRST® version. They were asked to report any difficulties in the understanding of the instrument or the instructions to fulfill the questionnaire; this step was performed by means of interview with each participant, who had to describe what they thought each of the sentences meant. All participants of this step considered the instrument understandable, and related to the FM symptoms they reported; no suggestions were made regarding words or sentences. After this test, the final Brazilian FiRST® was obtained.

For data collection, an initial assessment was designed to provide the sample’s demographical and clinical characteristics, including the following instruments: WPI, SSS, FiRST®, Numerical Rating Scale (NRS), Fibromyalgia Impact Questionnaire-Revised (FIQ-R®) and Brief Pain Inventory (BPI®). Seven to ten days after answering the questionnaires, participants were asked to answer again the translated version of the FiRST® for test-retest reliability and measurement errors. This period of time followed the COSMIN recommendations in which seven days after the test is the minimum time for the patients not to have changed in the measured construct and not to remember their answers to the instrument [11].

FiRST® is a screening tool for FM in patients with diffuse chronic pain [7]. It is a self-completed instrument composed of six items with answer options “yes” or “no”, in which the cut-off score of 5 points, which means that people who score 5 or 6 are likely to have FM. In the development study, FiRST® presented a sensitivity of 90.5%, a specificity of 85.7% (AUC = 0.93) and excellent test-retest reliability (ICC = 0.87) [7].

The NRS is a single item instrument that was used for pain and fatigue intensity. We evaluated pain in four different situations: at rest – “Currently and at the moment when you are sitting/lying on the couch watching your favorite TV show, do you feel pain?”; after body movement – “Currently and when you walked from the supermarket parking lot to the grocery store or crossed the street to work, do you feel pain?”; the lowest – “In the last week, what number corresponds to the most pain you have felt?”; and the greatest pain in the previous week – “In the last week, what number corresponds to the least pain you felt?” [12]. For fatigue, we asked “During the answer to this questionnaire, which number best corresponds to your state of fatigue/body tiredness?“. In all questions about pain/fatigue, zero means no pain/fatigue and 10 was the worst pain/fatigue imaginable. In chronic pain conditions, NRS had a moderate to high test-retest reliability (0.67 to 0.96) [13].

The FIQ-R® assesses the impact of FM on life in relation to functional capacity, professional status, psychological disorders and physical symptoms [14]. The Brazilian version of FIQ-R® had excellent test-retest reliability (ICC = 0.75) and comprises 21 items that investigate three domains: function (9 items, 30 points), global impact (2 items, 20 points) and symptoms (10 items, 50 points) [14, 15]. Scores range from 0 to 100, with the latter meaningful of a worst condition. The minimal important clinical difference for the FIQ-R® is 27 points [16].

The BPI® assesses pain severity and impact on a person’s life with 15 items that assess presence, severity, location, functional impact, used therapeutic strategies, and treatment efficacy in an 11-point scale ranging from zero (no pain/no interference) to 10 (as bad as it can be). High scores indicate worse pain severity and impact. The Brazilian version of the BPI® presented a two-dimensional structure (pain severity and interference) and excellent internal consistency (α = 0.87–0.91) [17].

Statistical analysis was performed using the Statistical Package for the Social Sciences (SPSS 26, IBM, USA). Gwet’s AC1 agreement was performed in RStudio. The characterization of the sample was represented by frequency (n, %), mean, and standard deviation (SD). To compare the FM and chronic pain groups, we used Mann-Whitney test, Chi-square and Fisher’s exact test.

To set a FiRST cutoff point for FM screening, we used the assessment by the ACR criterion to classify participants with and without FM. The area under the curve (AUC) of receiver operating characteristics (ROC) curve was used to determine sensibility, specificity, and cut-off scores to determine differences between chronic pain patients with and without FM diagnosis. The AUC ≥ 0.8 indicates excellent accuracy [18].

Test-retest reliability was assessed by Fleiss’ Kappa (κ), and measurement errors were calculated based on the percentage of agreement between the test and retest FiRST® scores. If there was a discrepancy between Fleiss’ Kappa and percentage of agreement, agreement was assessed using Gwet’s AC1 [19]. We considered the following values of κ, AC1 and percentage of agreement: κ and AC1 < 0.20 and percentage of agreement 0–4% were considered as “none”; κ and AC1 = 0.21–0.39 and percentage of agreement 4–15% as “minimal”; κ and AC1 = 0.40–0.59 and percentage of agreement 15–35% as “weak”; κ and AC1 = 0.60–0.79 and percentage of agreement 35–63% as “moderate”; κ and AC1 = 0.80–0.90 and percentage of agreement 64–81% as “strong”; and κ and AC1 > 0.90, and percentage of agreement 82–100% as “almost perfect” [19, 20]. The significance level of all tests was 5%.

## Results

The translated version of the FiRST® was tested by 10 healthcare professionals (mean age: 36.8 ± 12.7 years old; mean scholarity 17.7 ± 2.8 years; experience with Rheumatology patients: 14.1 ± 12.0 years) and 20 women with FM (age: 45.9 ± 9.2 years old; mean scholarity: 8.1 ± 5.7 years). Healthcare professionals did not suggest any changes to the sentences, and agreed on the translated version. No language adaptations were needed and the women did not present difficulties to understand the instrument. After that, the final Brazilian FiRST® was obtained.

For the evaluation of test-retest reliability, measurement errors and cut-off point, we received 573 answers during data collection period. Six people did not agree to take part in the study, 26 reported not feeling pain in the same intensity for at least 3 months (which mischaracterizes chronic pain), and 58 presented at least one of the exclusion criteria: not answering the FiRST® instrument (n = 1), reported schizophrenia (n = 2), reported borderline disturbance (n = 4), reported panic syndrome (n = 9), reported bipolar disturbance (n = 19), and reported severe depression (n = 23). As such, we had 483 valid answers, 395 with FM and 88 without FM. From them, 39 participants with FM answered the instruments in 7–10 days after the first evaluation. Table 1 shows the clinical and demographical characteristics of the participants.

**Table 1:** Sociodemographic and clinical characteristics of both groups

Variables	Fibromyalgia group (n = 395)	Chronic Pain group (n = 88)	p
**Age (years); mean (SD)**	40.7 (9.4)	43.8 (13.2)	0.061
**Weight (kg); mean (SD)**	74.4 (15.9)	72.8 (14)	0.557
**Height (m); mean (SD)**	1.62 (0.07)	1.63 (0.07)	0.860
**Sex (Women); n (%)**	389 (98.5)	83 (94.3)	0.049**
**Brazilian regions; n (%)** Southeast Northeast South North Central-West	193 (48.9) 110 (27.8) 43 (10.9) 25 (6.3) 24 (6)	53 (60.2) 16 (18.1) 10 (11.4) 3 (3.4) 6 (6.8)	0.236
**Marital Status; n (%)** Married Single Divorced Widow/widower	247 (62.5) 97 (24.5) 47 (12) 4 (1)	59 (67) 17 (19.3) 12 (13.6) 0	§
People sharing their house; n (%)* 1 to 3 people 4 or more	231 (60.8) 149 (39.2)	66 (76.7) 20 (23.2)	0.003***
People that depend on the participant regarding care; n (%)* None One person Two or more people	127 (33.5) 93 (24.5) 159 (41.9)	35 (40.7) 31 (36) 20 (23.2)	0.004**
**Scholarity; n (%)** Fundamental School High School Higher Education	24 (6.1) 109 (27.6) 262 (66.3)	5 (5.7) 23 (26.1) 60 (68.2)	0.946
Family income n (%) *missing values 0–3 minimum wages >3–6 minimum wages > 6 minimum wages	220 (62.1) 67 (18.9) 67 (18.9)	36 (50) 20 (27.8) 16 (22.2)	0.129
**Smokers; n (%)** Nonsmoker Smoker Former Smoker	353 (89.4) 22 (5.6) 20 (5)	81 (92) 1 (1.1) 6 (6.8)	§
**Medical license or retirement due to pain; n (%)** No Yes	282 (71.4) 113 (28.6)	76 (86.4) 12 (13.6)	0.002***
**Continuous use medication; n (%)** Yes No	319 (80.8) 76 (19.2)	62 (70.4) 26 (29.5)	0.025***
**Use of pain medication during crisis; n (%)** Yes No	346 (87.6) 49 (12.4)	66 (75) 22 (25)	0.003***
Health perception; n (%); *missing Very bad Bad Good Excellent	106 (26.8) 144 (36.5) 108 (27.3) 4 (1)	9(10.2) 19 (21.6) 50 (56.8) 8 (9)	§

*Missing values; **Chi-square test p < 0.05. ***Chi-square test; Fisher’s Exact test; p < 0.05.; § no statistical analysis was possible due to expected count < 5

Table 2 shows the scores of the NRS for pain and fatigue, the BPI® and the FIQ-R® for FM and chronic pain groups. The FM group had higher scores for all questionnaires, indicating a greater pain intensity, fatigue intensity, pain impact, and severity and FM impact (p < 0.001).

**Table 2:** Questionnaires’ scores for the participants of both groups, presented as mean (standard deviation)

Variables	FM group (n = 395)	CP group (n = 88)	p
NRS – Present pain at rest	6.8 (2.0)	5.0 (2.5)	< 0.001
NRS – Present pain after movement	7.6 (2.0)	5.4 (2.7)	< 0.001
NRS – Greatest pain in the previous week	8.6 (1.5)	7.0 (2.3)	< 0.001
NRS – Lowest pain in the previous week	5.4 (2.4)	4.3 (2.5)	< 0.001
NRS – Present fatigue	7.9 (2.1)	5.0 (3.4)	< 0.001
BPI – Pain severity	6.9 (1.6)	5.1 (2.3)	< 0.001
BPI – Pain impact	7.5 (2.0)	5.3 (2.8)	< 0.001
FIQ-R - Function (0–30)	21.4 (6.7)	11.5 (8.7)	< 0.001
FIQ-R – Global impact (0–20)	15.9 (4.4)	9.3 (6.4)	< 0.001
FIQ-R - Symptoms (0–50)	37.3 (7.4)	25.3 (12.4)	< 0.001
FIQ-R – Total score (0-100)	74.6 (16.6)	46.1 (25.6)	< 0.001

FM: Fibromyalgia group; CP: Chronic pain group; NRS: Numerical Rating Scale; BPI: Brief Pain Inventory; FIQ-R: Fibromyalgia Impact Questionnaire-Revised.

**Fig. 1:**
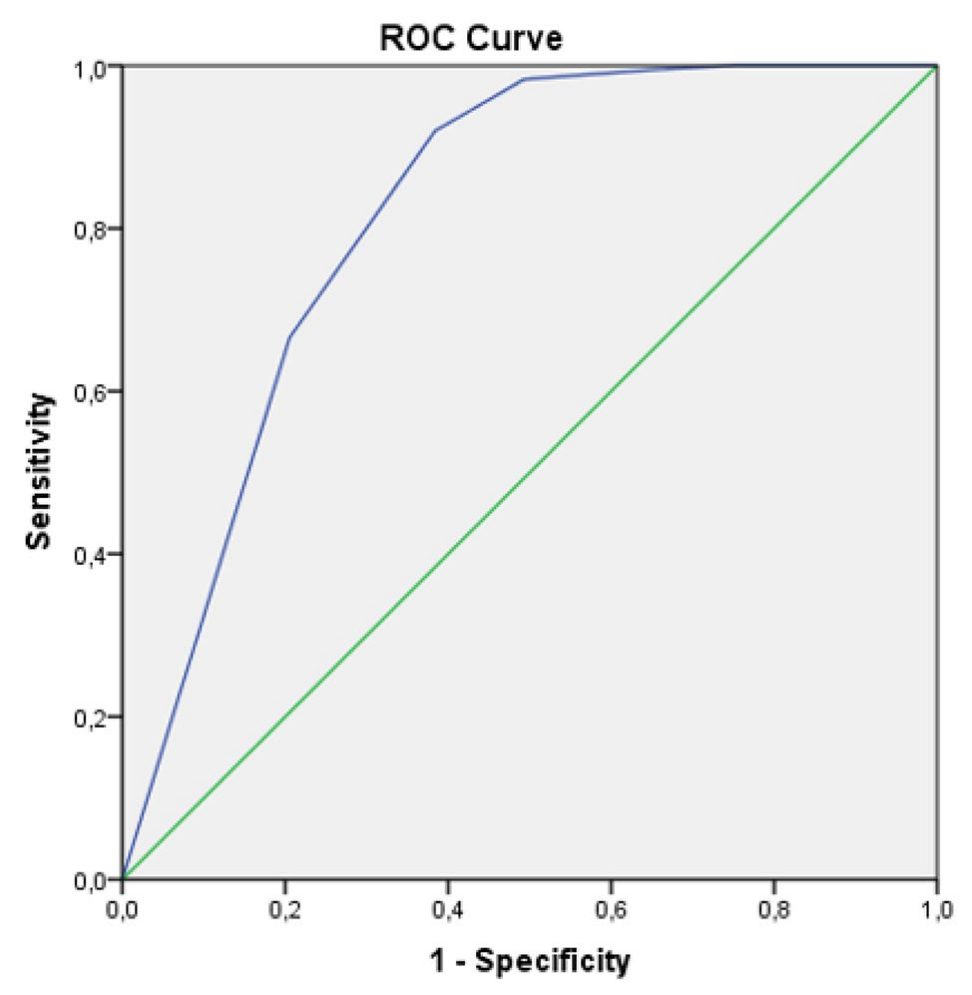
– Receiver Operating Characteristics curve for the Brazilian version of the FiRST instrument

The ROC curve (Fig. 1) indicated a sensitivity of 92.3% and a specificity of 61.6% with a cut-off score of 4.5. Considering that the FiRST® only scores whole numbers, the cut-off score can be set at 5, as the original instrument. The Positive Predictive Value was 93.4% and the Negative Predictive Value, 57.7%; the positive likelihood ratio was 2.47, while the negative likelihood ratio was 0.12. The Area Under the Curve (AUC) was 0.819 (95%CI 0.754–0.883, SE 0.033), indicating a good ability to distinguish people with FM from people with chronic pain without FM. Table 3 shows the agreement of participants with and without FM by the 2016 ACR and the FiRST®. Among the 395 people with FM by the 2016 ACR, 357 (90.4%) were also classified with FM and 38 (9.6%) without FM by the FiRST®.

**Table 3:** Agreement between FiRST and 2016 American College of Rheumatology fibromyalgia screening

	2016 ACR with fibromyalgia	2016 ACR without fibromyalgia	Total
**FiRST – with fibromyalgia**	357	43	400
**FiRST – without fibromyalgia**	38	45	83
**Total**	395	88	483

FiRST: Fibromyalgia Rapid Screening Tool. 2016 ACR: American College of Rheumatology

For the test-retest reliability, FiRST® had a minimal value [κ = 0.38 (95% CI 0.15–0.62); p = 0.001]. Gwet’s AC1 showed moderate agreement between the test and retest [AC1 = 0.63 (95% CI 0.42–0.84)]. For the measurement errors, the percentage of agreement between test and retest was strong (71.8%), and of disagreement was 28.2%.

## Discussion

The present results show that the Brazilian online version of the FiRST® was successfully translated, and presented a good content validity and adequate errors measurement. Differently from the other versions, this was the first to be applied online and to consider the 2016 ACR diagnostic criteria. Also, it was possible to observe that the FM group presented higher scores for all questionnaires applied, which means a worst condition.

FM has a clinical diagnosis [2] and it usually takes years to be diagnosed and treated [21], as such, a more rapid diagnosis would imply in a sooner intervention [22], which could prevent patients from suffering long-term consequences of their pain and symptoms. Along with the changes in the way one diagnosis FM through the years [3, 23, 24], instruments that allow healthcare professionals to screen for FM in different clinical settings makes this diagnostic process faster. In this sense, the FiRST® was one of the first instruments with this proposal, and has been translated into several languages, such as French [25], Spanish [26, 27], Greek [28] and Turkish [29].

The online Brazilian FiRST® obtained similar results regarding specificity, sensitivity, positive and negative likelihood ratios as the other translated versions. Zis et al. [28] obtained a sensitivity of 86% and a specificity of 83% for the cut-off score of 5 points, with a positive likelihood ratio of 4.97 and a negative likelihood ratio of 0.17, using both ACR 1990 [23] and 2010 [24] as diagnostic criteria for FM patients, and compared them with patients with osteoarthritis. Torres et al. [26] used the ACR 1990 for FM patients and compared them to patients with other chronic pain conditions such as arthrosis and polyneuropathies, and obtained a sensitivity of 89% and a specificity of 55.3%, with a positive likelihood ratio of 1.99 and a negative likelihood ratio of 0.20 for the cut-off score of 5 points. Celiker et al. [29] used the ACR 2013 modified diagnostic criteria [30], and for the cut-off score of 5 points, obtained a sensitivity of 83.8% and a specificity of 68.4%, with a positive likelihood ratio of 2.65 and a negative likelihood ratio of 0.24. The Brazilian FiRST®, when compared with the other versions, obtained similar values of sensitivity, specificity, positive and negative likelihood ratios, even with the data collection occurring in an online manner. Online patient assessment reduces evaluation time, and turns it easier for professionals to access data as well as to compare patients’ pre- and post-intervention status. Also, data cloud storage prevents data from being lost, as well as reduces the number of paper files that need to be stored for years. For the patient, sometimes not being in front of a healthcare professional turns it easier to report things that are considered taboo, such as substance abuse or suicidal ideation.

Regarding diagnostic criteria for FM, two scales are currently used (WPI and SSS) proposed by Wolfe et al. in 2011 [31], updated in 2016 [3], and adapted for telemedicine in the Brazilian population in 2019 [32], whose reliability and internal consistency have already been established (κ > 0.6; Cronbach’s alpha > 0.73). This occurred because the authors verified the criteria for the diagnosis of FM presented in previous classifications (such as the use of trigger points) should focus more efforts on the patient’s self-report via three observations: (1) presence of pain in at least four of the five body regions; (2) presence of symptoms with similar severity for at least three months; (3) absence of chronic conditions and/or diseases that justify the symptoms - we confirmed these criteria in all patients who had a clinical diagnosis of FM.

A reliable tool to screen for FM can turns it easier for professionals to suspect on the diagnosis and provide better healthcare in all levels of attention. This is particularly valuable considering the Primary Health Care, usually the entrance door through which one individual assesses the healthcare systems. By working in a biopsychosocial model of attention to chronic pain [8], such as FM, it is possible to better articulate with all professionals that are part of the staff who could provide attention to the patient that is likely to have FM diagnosis. Even if the instrument has a number of false negatives, it is possible to treat him/her into the fibromyalgianess concept [22] and provide care aiming to diminish the impacts of a long-term exposure to chronic pain.

After the COVID-19 pandemics hit the world, healthcare professionals had to learn new strategies to assess and treat patients with chronic pain [33] and online assessment instruments became popular and much more used since then. The Brazilian version of the FiRST® was validated to be applied online, which can turn the screening process even easier and faster, allowing any healthcare professional to identify and refer those patients more adequately. Although it has limitations in terms of scope, the advent of validation of online instruments is a scientific and technological advancement in the health area, because it guarantees the simultaneous screening of several patients and optimization of professional’s time [32].

The present study has some strengths; the online data collection allowed people from all over the country to take part in the study, and they had a chance to answer the Google Forms from wherever they were, giving them a chance to complete the forms when they were most comfortable, without the need to travel to a certain location for data collection. Also, the online validation may serve as a way of implementing this instrument as a prior patient’s assessment, in a manner that the patient can go to the healthcare facility already screened for FM. However, there were some limitations of the present study as well; for example, regarding the online data collection, which did not allow researchers to assess participants and confirm their diagnosis. This form of data collection prevents participants to clarify possible issues regarding their comprehension of the questions. Likewise, only people with access to internet and an e-mail account could take part in the study, as well as only people who could read. This is an important issue as it does not reflect the Brazilian reality, in which 21% of the Brazilians do not have access to internet [34] and about 11% of people ≥ 40 years old and about 18% of people ≥ 60 years old are illiterate [35].

## Conclusion

The FiRST® was translated into Brazilian Portuguese and the online version presented a good content validity and adequate errors measurement that allow FM patients to be screened with a cut-off score of 5 points.

## Data Availability

The datasets generated and analysed during the current study are not publicly available due to personal content that can lead to participant identification but are available from the corresponding author on reasonable request.
